# Image analysis of weaverbird nests reveals signature weave textures

**DOI:** 10.1098/rsos.150074

**Published:** 2015-06-17

**Authors:** Ida E. Bailey, André Backes, Patrick T. Walsh, Kate V. Morgan, Simone L. Meddle, Susan D. Healy

**Affiliations:** 1 School of Biology, University of St Andrews, Harold Mitchell Building, St Andrews, Fife KY16 9TH UK; 2 Faculdade de Computação, Universidade Federal de Uberlândia, Avenida João Naves de Ávila, 2.121—Campus Santa Mônica, UBERLÂNDIA, Minas Gerais CEP 38400-902, Brasil; 3 School of Biological Sciences, University of Edinburgh, Ashworth Laboratories, King's Buildings, Mayfield Road, Edinburgh EH9 3JT, UK; 4 Roslin Institute, The Royal (Dick) School of Veterinary Studies, University of Edinburgh, Easter Bush, Edinburgh EH25 9RG, UK

**Keywords:** texture analysis, nest building, biological structures, classification, individual differences

## Abstract

In nature, many animals build structures that can be readily measured at the scale of their gross morphology (e.g. length, volume and weight). Capturing individuality as can be done with the structures designed and built by human architects or artists, however, is more challenging. Here, we tested whether computer-aided image texture classification approaches can be used to describe textural variation in the nests of weaverbirds (*Ploceus* species) in order to attribute nests to the individual weaverbird that built them. We found that a computer-aided texture analysis approach does allow the assignment of a signature to weaverbirds' nests. We suggest that this approach will be a useful tool with which to examine individual variation across a range of animal constructions, not just for nests.

## 1. Introduction

Famous artists and architects are noted for the individually characteristic style of their creations. Based on the style and techniques used in its creation, a painting can be assigned not only to an individual artist but also to the time in the artist's career at which it was painted [1]. Individual animals also create structures, such as the caddisfly's Trichoptera case [2], the puffer-fishes' *Torquigener* sp. metre-wide, geometrically patterned nests [3], the decorated bowers of bowerbirds Ptilonorhynchidae [4], the lodges and dams of beavers Castoridae [5] and the woven nests of weaverbirds Ploceidae [6].

Examination of the nests built by weaverbirds and sticklebacks reveals that, like humans, they may also exhibit individual differences in the structures they create, at least in terms of the structure's gross morphology, e.g. mass, number of pieces of material added, shape, length, height, volume and so on [7,8]. Consistent individual differences in behaviour (repeatability) are of growing interest because they may correlate with fitness and appear to be heritable in some instances [9–12].

Identifying repeatability in the form of a construction is a first step in testing whether all individuals of a species produce the same structure in the same way and if not, for exploring whether genetic or behavioural explanations are more likely to underlie any observed variation. Until now, attempts to quantify ‘individuality’ or variability among individual animal constructions have been limited to measures of gross morphology or to quantification of elementary building behaviours [3,8,13–17]. The morphology of an object is, however, more than just its gross measurements, but is a combination of size, shape, colour patterns and texture [18]. Although measuring the size and shape of objects in a consistent and repeatable way is fairly straightforward, the same is not true of texture, a property that can allow us to describe the finer scale attributes of an object, which may relate more closely to the method of its construction than does gross morphology [19]. Furthermore, the texture of a biological construction may relate to important physical properties such as imperviousness to wind and rain.

Surface texture is a property of most surfaces that contains information about the structural arrangement of surface elements [20]. Textural features result from the spatial distribution of tonal variation within a band of the electromagnetic spectrum, where tone is visualized as varying shades of grey in a black-and-white image [20]. Textural features include image characteristics such as the consistency of grey tone in the image, the size, curvature and orientation of linear structures, the density and hardness of boundaries between areas of different grey tone or coarseness, and contrast [20]. While ad hoc methods for examining selected components of texture such as the frequency and degree of loose loops protruding from the surface of a nest, or the density of weaving (fibres per centimetre), might be useful, they cannot fully capture all the information that may be contained within textural features [21,22]. Given the potential importance of the surface integrity of biological constructions to the builder's reproduction and survival, it would be useful to identify methods that can be used to quantify texture in a reliable and repeatable fashion.

The rise of computer-aided image texture analysis offers a unique opportunity not only to measure these biological structures in more detail than has been achieved previously, but it also allows for investigation of the repeatability of fine-scale patterns created on the surface of structures by individual builders [23]. This is because texture analysis can help uncover what, to the human eye, may be otherwise undetectable variation among groups of objects in their surface texture. Such technology permits much greater resolution than achieved with other methods and has led to accurate identification of cryptic plant species by leaf shape and pattern [24,25] and of the population of origin of spring salamanders *Gyrinophilus porphyriticus* [26,27]. Multiple methods of computer-aided texture analysis have been developed, many of which are fine-tuned to work using specific textural features. This allows users to choose to use one or a combination of methods that are specifically useful for analysing the features that are present in their images [20,28]. Assuming that there is sufficient consistency in texture within and variation among the categories of interest, texture analysis can be used to attribute objects to the correct category.

The nests woven by individual weaverbirds represent an ideal structure with which to test the value of image texture analysis for investigating individual variation, i.e. their woven signature, in animal-built structures. Firstly, texture analysis using computer vision has already proved to be a useful method for classifying fabrics woven by humans [29]. Secondly, while there is evidence to suggest that in some species (southern masked weavers, *Ploceus velatus*), there is repeatability in the overall dimensions (e.g. length and width) of the nests built by individual weaverbirds, in other species (village weavers, *Ploceus cucullatus*), such repeatability is not apparent [8]. It is possible that gross measures of nest morphology may mask repeatability at finer scales of construction for the nests built by village weavers, but that repeatability may be apparent in weave texture. Thirdly, it is conceivable that individual differences in morphology or experience in choosing, cutting and weaving material into a nest will result in consistent individual variation in the surface texture of the nests they build.

In this study, we used computer-aided textural analysis of digital photographs from multiple nests built by known male village and southern masked weaverbirds to: (i) explore its potential as a method for identifying the individual weaverbird builder; and (ii) identify which classification methodology works best for weaverbird nests.

### 1.1 Predictions

There were three possible outcomes when investigating the accuracy with which texture classification approaches will correctly attribute nests to the male weaverbird that wove them: (i) below chance, (ii) above chance, and (iii) very high-classification accuracy (see table 1 for predictions).

**Table 1.** Possible interpretation of computer-aided texture analysis methods of the different levels of classification accuracy (attribution of a nest to its builder) that could be achieved from the surface texture of weaverbird nests.

classification accuracy	possible interpretation of computer-aided texture analysis methods
chance or below	cannot be used to attribute nests accurately to the male weaverbird that built them even though there is high within-individual consistency in weave pattern
	there is no significant within-individual consistency in birds' weave pattern
above chance	can be used with only limited accuracy to attribute nests to the male that built them despite high within-individual consistency in weave pattern
	are very effective for attributing nests to the male that built them, but there is very limited within individual consistency in weave pattern (only some males are consistent or all males are moderately consistent)
very high	are very effective for accurately attributing nests to the male that built them and there is high within-individual consistency in weave pattern

## 2. Material and methods

### 2.1 Nest collection

We collected the completed nests of six village weavers (*P. cucullatus*) in June and July 2008, from Laminga Village, Jos, Nigeria. A total of 23 nests were collected, giving us two to five nests per male (mean: 3.83±0.48 nests). We also collected completed nests of southern masked weavers (*P. velatus*) from Botswana (Atholl Holme 11-KO, Gaberone) from 15 males in November and December 2008, and from seven males in November and December 2009 (we had collected nests from four of these seven males from the previous year and nests from three new males, leading to a total of 18 males). Ninety-six nests were collected in total (two to 13 nests per male; mean: 5.33±0.70 nests).

### 2.2 Nest photography

**Figure 1.**
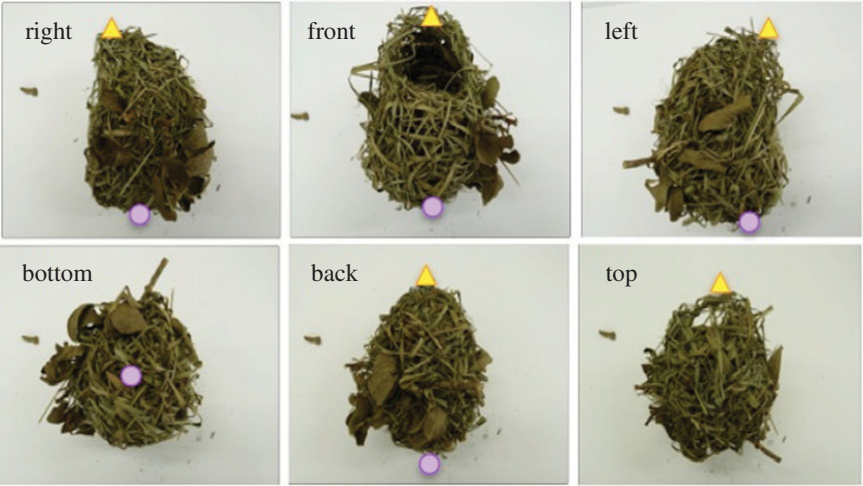
Photographs of all six faces of one village weaver nest. In the wild, nests are built so that the entrance faces down. The yellow triangles mark the entrance tunnel and the violet circles the back wall of the nest chamber.

Photographs of village weaver nests were taken in the field in 2008 or 2009 at or close to the time of collection against a white background, from a distance of 1 m, using a Canon powershot S5IS 8 mega pixel camera with auto focus. As these nests were then discarded, they were not available to this study. The nests of the southern masked weavers were kept dry and stored at room temperature before being photographed for this study in 2012. Photographs of these nests were taken from a fixed distance of 50 cm using a Panasonic Lumix DMC-FZ45 16 mega pixel camera with auto focus. Images of at least five sides of the nests were captured for all nests: front, back, top, bottom and one lateral side (figure 1).

### 2.3 Nest datasets

The nest images were grouped into four different databases according to species and year (table 2). The nests of males that had built only a single nest in the time period of interest were excluded.

**Table 2.** A summary of the contents of, and texture analysis classification accuracy in, each of the four weaverbird nest datasets. (For each dataset: the number of male weaver birds (all birds had multiple nests), the total number of nests, the mean number of nests per male weaver bird, the chance of attributing a nest to the male that built it at random, the percentage of the 465 possible classification approaches that performed better than chance, the performance of the classification approach that achieved the highest classification accuracy across all datasets and the classification success of the classification approach that achieved the highest classification accuracy in that dataset. For the most accurate classification approach in each dataset, the nest images and texture analysis methods that approach included: DCT, discrete cosine transform; WT, wavelet transform; GF, Gabor filter; VFD, volumetric fractal dimension.)

						texture analysis approaches				
dataset							most accurate approach over all datasets	most accurate approach (dataset specific)		
species	year	no. males with multiple nests	total no. nests	nests per male ($\bar{x}\pm s.e.$)	chance of correctly attributing a nest to a male at random (%)	approaches performing above chance (of 465) (%)	classification success (%)	classification success (%)	nests images used	analysis methods used
village	2008	6	23	3.83±0.48	16.67	33	43.48	69.57	back, front	DCT, WT
masked	2008	14	72	5.14±0.59	7.14	57	19.30	33.33	back, bottom, top	DCT, GF, WT
masked	2009	7	23	3.29±0.29	14.29	39	63.34	81.82	bottom	VFD
masked	2008+2009	18^a^	96	5.33±0.70	5.56	52	15.28	28.57	top	WT

^a^There are 18 rather than 21 males in the masked weaver 2008/2009 dataset because three males that built multiple nests both years appear in both 2008 and 2009 datasets. One male only built one nest in one of the years but multiple nests in the other year such that the single nest is only included in the combined dataset making 96 rather than 95 nests in total.

To create databases where all nests were described by the same image set, we used the back, bottom, front and top images for each nest [30]. Photos of the lateral side between years were not consistently of the same side, so were not included in the analysis.

### 2.4 Pre-processing of nest image

**Figure 2.**
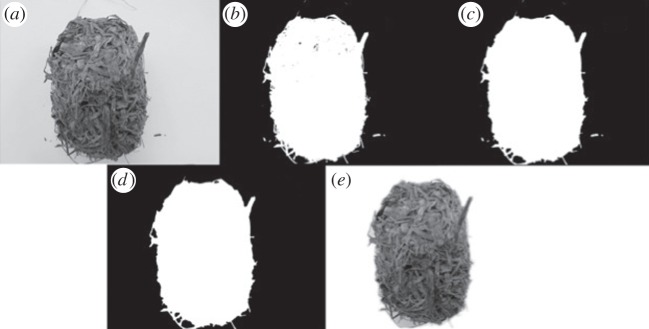
Image processing steps to remove small objects and background. (*a*) Original image; (*b*) binary image; (*c*) image after closing operation; (*d*) removing of all connected components from the binary image associated to noise; and (*e*) original image without noise and background.

Before the nest images were analysed, it was necessary to process the images to guarantee quality. We discarded all colour information from the nest images by converting all images to grey scale using luminance (figure 2*a*), using the following equation:

L=0.2126×R+0.7152×G+0.0722×B,

where *L* is the luminance and *R*, *G* and *B* are, respectively, the red, green and blue channels of the image.

The image background was subsequently removed as any variations in illumination could have been mistaken as a meaningful characteristic in the analysis. This was achieved with a threshold operation, which results in a binary image (figure 2*b*). The threshold operation converts a grey-scale image to a binary image by considering all pixels whose value is above a given threshold as white and the remaining pixels as black. Next, a closing operation was applied to remove holes present in the interior of the nest images (‘binarization’; figure 2*c*). The closing operation was performed by the dilation of the binary image followed by an erosion using the same structuring element for both operations, so that all small apertures in the image were closed. Then small objects present in the images, which were not related to the nest structure, were digitally removed to reduce variability during the analysis. To do this, all connected components (clusters of similarly coloured pixels, i.e. the small white patches in figure 2*c*) were removed from the binary image except the one representing the nest, i.e. the one with the largest area (figure 2*d*). The resulting filtered binary image was then combined with the original image, so that the original image was without noise or background (figure 2*e*).

Image processing and subsequent texture analysis were performed using Matlab 2014 on a desktop computer with an Intel Core 2 Quad Q8400 CPU (2.67 GHz), 4 GB of RAM, running Windows 7 (64 bit).

### 2.5 Texture analysis

Texture analysis uses a combination of different textural descriptors to classify images. One example of an image descriptor is the probability of adjacent pixels having the same grey-scale values over different distances and in different orientations [31]. When using texture analysis to classify three-dimensional objects, it is important to note that images of different faces of an object may contain different textural information, and therefore the images of some of those faces may be more useful for classifying that object (e.g. a nest) than are others. Therefore, it is useful to determine which faces provide the most useful textural information for any given dataset and use only those images.

Furthermore, descriptors calculated using different texture analysis methods, or combinations of methods, may vary in the degree to which classification is accurate depending on the dataset. Because the nests had been built by different species and/or in different years and were probably then built under different environmental conditions, we analysed them as separate datasets (split by species and year). Therefore, we sought a bespoke classification approach (set of descriptors for describing texture in images of the most texturally informative faces of an object) for each dataset as well as the best overall approach for classifying nest texture.

### 2.6 Texture descriptors

To identify which textural descriptors were the most informative when classifying nest texture, we computed a set of image descriptors (data variables) for each image to be classified, using up to five different methods. We chose the five methods based on their success in other contexts (classification of coral-reef images: [32], fabric defect detection: [33], e.g. tree bark species identification: [34], plant leaf identification: [35]) to maximize the likelihood that one of these methods would prove useful for classifying nest weave texture. For each dataset, we then tested the value of a variety of different combinations of textural descriptors for classifying nest images. The most informative descriptors for image classification were then selected using principal component analysis (PCA). The five texture analysis methods used are as follows.

(i) Discrete cosine transform (DCT): this method converts image data (i.e. their pixel values) into frequency data by compressing highly correlated neighbouring pixels in an image into fewer decorrellated parameters [36]. The input image is split into a number of sub-blocks (e.g. 8×8 pixel areas). DCT is then applied to the value of each pixel in the block to produce an 8×8 matrix of frequency coefficients. The value of the pixel in the top left of each block is used as a reference and the coefficients for other pixels in the block are calculated relative to it. A single value is then calculated for each block by convolution of a predefined mask (i.e. a set of weight values associated with each position of the block). A single image descriptor for each image and mask is then calculated as the sum of the calculated values of the blocks. We computed a total of nine descriptors per image using nine orthogonal masks derived from the discrete cosine transform [37].
(ii) Gabor filter: this method is based on spatial frequency analysis and is designed to mimic the band-pass filter bank properties of human vision. A band-pass filter restricts which frequencies of an input signal are used in the calculation of an output signal. An example of a frequency in image processing would be the spacing of horizontal stripes. The output signal for stripes running in different orientations could, therefore, be very different. Gabor filtering uses a Gaussian function to determine which frequencies are used to calculate the output signal or (texture signature). We used a total of 24 Gaussian functions to capture different orientations and scales of texture in the nests' weave patterns (six rotation filters at four different spatial scales). These filters considered frequencies from 0.05 to 0.4. We performed the convolution of each Gabor filter over the input image and then computed the energy (i.e. the sum of the squared convolution values) as the descriptor for each of the 24 Gabor filters [38].
(iii) Two-dimensional multilevel wavelet transform: the basic premise of this method is to represent functions used to describe image features as a series of wavelets, i.e. wave-like functions built under specific parameters [39]. The method performs the convolution of this wavelet function over each row and column of an input image (dyadic decomposition). This process results in four new images, each one with half the size of the original image: one image (the approximation) represents the main aspects of the original image (low-frequency information), while the other three are coefficients representing the image details (high-frequency information) in different directions (horizontal, vertical and diagonal). This process can be repeatedly applied for each computed approximation and so forth. From the details of the resulting images, we can calculate energy, entropy and mean. In total, we calculated 27 descriptors for each image using Daubechies 4 (a family of orthogonal wavelets) via three levels of dyadic decompositions [40,41].
(iv) Tourist walk: this approach considers each pixel as a city in a map. From each city/pixel leaves a tourist wishing to visit cities (other pixels) according to the rule of going to the nearest (or farthest) city that has not been visited in the last *μ* time steps. The path taken depends on the difference in intensity between pixels. The tourist stops when they enter a cycle of pixels from which they cannot escape. At the end, each tourist trajectory consists of a transient path of length (new cities are visited) and a final cycle period where new cities are not visited any longer. From these trajectories, it is possible to build a joint distribution of the transient time cycle period of the image. We extracted four values from this joint distribution (called walking histogram). In total, we calculated 48 descriptors for each image using two rules (nearest and farthest) and six different *μ* values according to the specification in [42].
(v) Volumetric fractal dimension: this method considers an image as a surface by mapping each pixel as a point in three-dimensional space where the additional dimension is the value (e.g. colour intensity) of the pixel. We computed the influence volume for a specific radius *r*. The influence volume is very sensitive to structural changes of the texture in the image. Basically, it is the number of points in the three-dimensional space whose Euclidean distance to the image surface is smaller or equal to *r*. We considered the volume computed for *r*=[1,=*v*] as the descriptors used to describe the image, which give us a fine-to-coarse representation of the image [27].

Each method resulted in a specific number of descriptors. It is important to emphasize that more descriptors do not guarantee that a method is more accurate as the number of descriptors is usually defined by the method's authors. While some methods have the number of descriptors defined mathematically, others are empirical. Moreover, each method explores the data provided by an image in different ways. This can make one method better than another for some image patterns but it does not guarantee that this method will then be more accurate than another across a range of image patterns.

In total, 128 descriptors were calculated across all five methods for each image. In reality, only a few of these descriptors were used in image classification as most were excluded using PCA while still maintaining 95% of the discriminatory power. Analyses were run blind to male identity.

### 2.7 Texture classification approaches

Here, we define a classification approach as a combination of images of each nest (e.g. top and bottom) and texture analysis methods/descriptors (e.g. tourist walk and Gabor filter) that can be used across a dataset to attribute the nests in that dataset to the bird that built them. For each nest dataset, we evaluated classification accuracy for all possible combinations of the five texture analysis methods tested.

The five texture analysis methods can be combined in 31 different ways: each on its own, all possible pairs, all possible groups of three, all possible groups of four and all five methods.

As we did not know which face of the nest provided the best information for its characterization, we evaluated all possible combinations of all four faces of the nest: back, bottom, front and top. These four faces can be combined in 15 different ways.

Therefore, descriptors could be calculated for 31 different combinations of texture analysis methods paired with 15 possible combinations of nest faces resulting in 465 possible analysis approaches (combinations of nest face/texture analysis methods) to be evaluated for each dataset of nest images.

**Figure 3.**
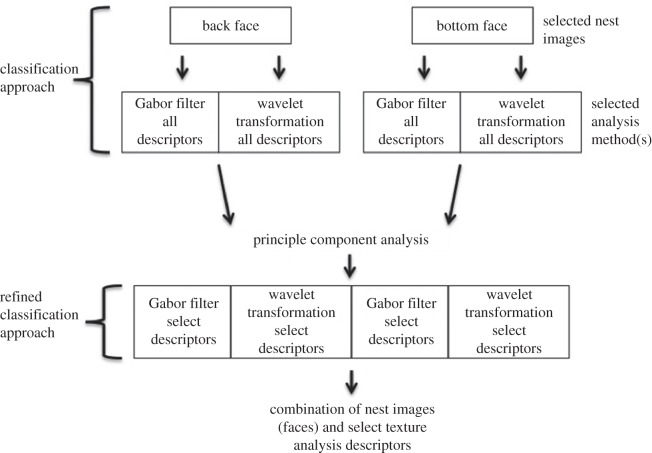
The process of refining the texture descriptors considered in a classification approach.

The best textural descriptors, for all possible nest face and texture analysis method combinations, were identified as follows: given an individual descriptor or combination of descriptors (e.g. Gabor filter and wavelet transform) and a face or multiple faces (e.g. back and bottom), we computed the selected descriptors (PCA features) for each image face using the texture analysis methods described above. Then, we concatenated them into a single set of descriptors as shown in figure 3.

We assessed the classification accuracy of different approaches using the statistical classifier linear discriminant analysis (LDA). This classifier considers that all the classes (male weaverbirds) have the same covariance matrix and separates them by hyperplanes [43,44]. We also used a leave-one-out cross validation scheme, a common approach to estimate the classifier error [45]. In this approach, each male's nest was compared to all remaining nests in the dataset in order to find which was the most similar. If the most similar nest belonged to the same male, we considered it as successfully attributed. Success rate was defined as the percentage of nests successfully attributed to their respective males.

The classification accuracy of approaches using LDA was further evaluated by comparing it to chance. Chance represents the probability that a given nest is associated with the correct male at random and was calculated as

$$\mathrm{chance}=\frac{1}{\text{no. males with multiple nests}}.$$

Complete classification failure (e.g. unable to classify; UC: table 3) meant that the textural descriptors produced a covariance matrix for which it was impossible to compute its inverse. This problem results from bad conditioning of the data (e.g. a matrix with determinant equal to zero, or where its columns are not linearly independent) used in the classifier.

**Table 3.** Rows contain information on the classification accuracy of different classification approaches in a given dataset and the columns represent the different classification approaches. (These classification approaches are those we found to have the highest classification accuracy in specific datasets (dataset of origin). The dataset of origin of each classification approach is indicated in the column headings. The classification accuracy of each approach in its dataset of origin is highlighted in italics. UC, unable to classify.)

			most accurate approach for			
dataset	chance (%)	best overall approach (%)	village 2008 (%)	masked 2008 (%)	masked 2009 (%)	masked 2008+2009 (%)
village 2008	16.67	43.48	*69*.*57*	UC	UC	UC
masked 2008	7.14	19.30	17.24	*33*.*33*	14.04	26.23
masked 2009	14.29	63.34	18.18	UC	*81.82*	27.27
masked 2008+2009	5.56	15.28	4.05	8.33	UC	*28.57*

## 3. Results

### 3.1 Overall classification success

In this section, classification approaches are described with regard to their correct attribution of nests to their builder.

**Figure 4.**
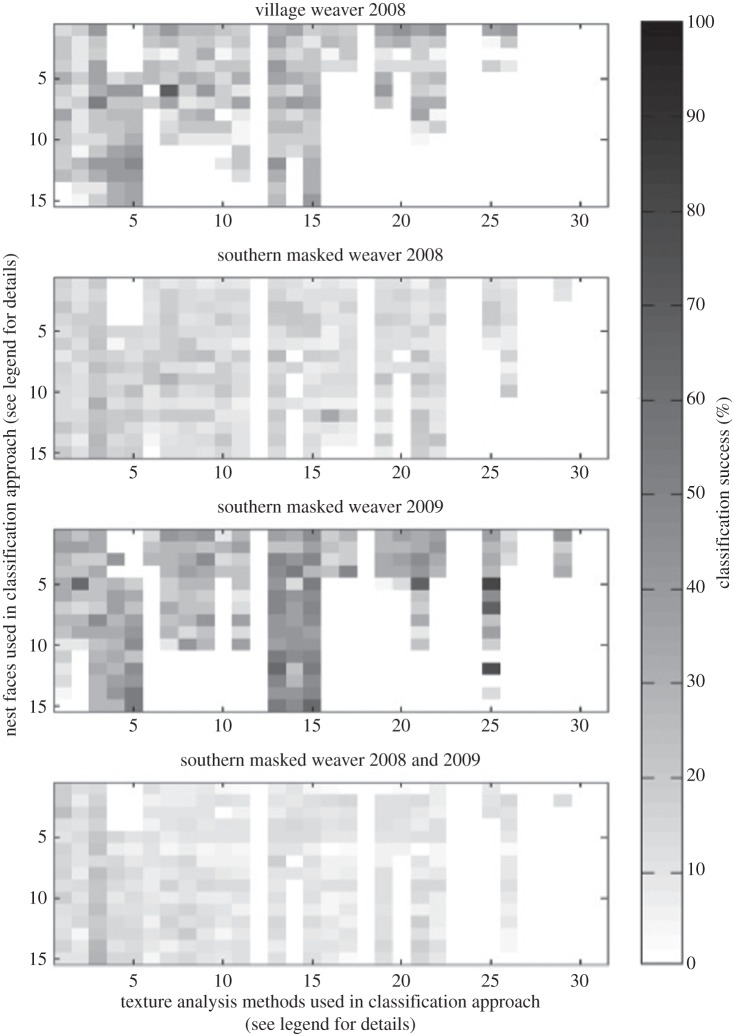
The classification accuracy in each of the four datasets of all 465 possible classification approaches. Each tile represents a different dataset and each cell in the tile represents one of the 465 classification approaches tested. Rows of cells represent the nest face(s) used in the analysis approach: rows 1 to 4=single nest face, rows 5 to 10=two nest faces, rows 11 to 14=three nest faces, row 15=four nest faces. Columns of cells represent the analysis methods/combination of methods used in the analysis approach: columns 1 to 5=single texture descriptor, columns 6 to 15=two analysis methods, columns 16 to 25=three analysis methods, columns 26 to 30=four analysis methods, column 31=five analysis methods. The classification success (%) of each analysis approach is indicated by the darkness of its pixel. Zero (white)=unable to classify.

There was considerable variation in classification accuracy of different classification approaches (combination of faces/descriptors) in the different datasets (figure 4). On average, across all datasets, 45.25 (±5.57 s.e.)% of the 465 analysis approaches tested performed better than chance (see table 2 and figure 4). When examining each of the 465 analysis approaches individually, 66% performed better than chance on at least one dataset, 23% performed better than chance in all four datasets, 15% on three of the datasets, 19% on two of the datasets and 9% on a single dataset.

Of the classification approaches that appeared to be more accurate than chance across all four datasets, the best (table 2) was, on average, better than chance by 24.43 (±9.03)% but only tended to be more accurate (paired *t*-test: *t*_3_=2.69, *p*=0.07). This best overall approach used the wavelet transform+tourist walk texture descriptors and images of the back, bottom and top of the nests.

### 3.2 Best classification approaches for each dataset

**Figure 5.**
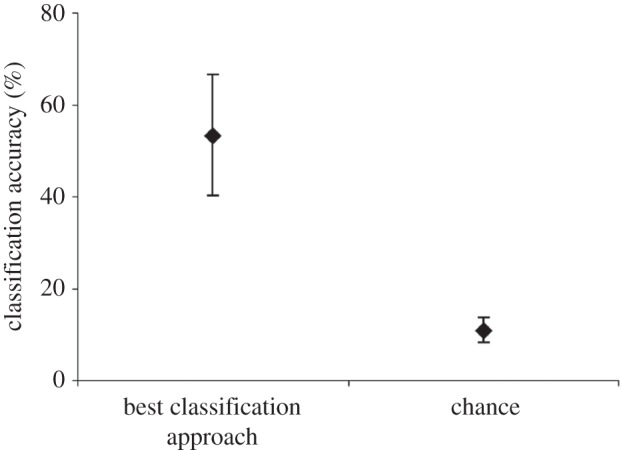
The classification accuracy (% of nests successfully attributed to the male that built them) achieved by: (a) assigning nests to males using the best texture analysis approach found for each dataset, (b) assigning nests to males at random (chance).

For each of the datasets, other approaches worked better than the best overall approach. The best classification approach for each individual dataset correctly assigned nests to the males that built them with an accuracy ranging from 28.57 to 81.82% (table 2). Furthermore, these best classification approaches performed better than chance by 23.01 to 67.53% (paired *t*-test: *t*_3_=3.95, *p*=0.03; figure 5).

Classification accuracy, based both on Akaike's information criterion (AIC) and effect size, was better predicted and more strongly affected by the number of nests/male within the dataset (adjusted *r*^2^=0.99, effect size=−26.51±0.62%, AIC=15.22, *F*_3_=1571.58, *p*<0.01), than it was either by the number of males within a dataset (adjusted *r*^2^=0.83, effect size=−4.33±1.08%, AIC=33.54, *F*_3_=16.21, *p*=0.06), or the number of nests within a dataset (adjusted *r*^2^=0.89, effect size=−0.69±0.14%, AIC=31.98, *F*_3_=25.55, *p*=0.04; table 2).

We examined the performance of the most accurate classification approach for each dataset when applied to other datasets (table 3). The best classification approach for each focal dataset tended not to perform as well when it was applied to other datasets, if at all (classification accuracy for the focal dataset compared with the mean classification accuracy for other datasets; paired *t*-test: *t*_3_=3.04, *p*=0.06; with complete failure to classify in 42% of ‘other’ datasets; table 3).

## 4. Discussion

Here, we demonstrate that we can use computer-aided image texture analysis to correctly assign weaverbird nests to an individual builder. This marks a useful step forward in our ability to determine firstly, variability in otherwise apparently similar biological constructions and secondly, to determine individuality in those constructions.

Although the texture analysis can be used to classify nests according to the male that built them at above chance levels, classification accuracy was short of 100% (varying from 28.57 to 81.82%). There are two possible reasons for the lack of a consistently high degree of classification. The first is one of methodology: the texture analysis methods that we used were limited in their accuracy in attributing nests to their builder even if there was high within-builder consistency in weave pattern, because, for example, they were just not appropriate for classifying the sort of texture typical of weaverbird nests. The second is biological. If male weavers are only partially consistent in their weaving patterns, no texture classification approach would be able to achieve very high-classification accuracy. While it is impossible for us to say at this time which of these interpretations is most likely to be correct, it is clear that texture analysis can be used to attribute animals' constructions to their builder, with up to 81.82% accuracy in the case of these weaver nests, and that there is at least some identifiable individual consistency in weaverbirds' weave patterns.

Not only does there appear to be an identifying texture to the weave of an individual's nest, male weaverbirds also appear to be more consistent in their weave pattern than they are in the size and shape of the nests they build. For example, Walsh *et al.* [8], who used largely the same nests as we used in this analysis, found that gross nest morphology was not repeatable within male village weavers. This may be because measures of gross nest morphology are too coarsely grained to capture slight but significant variation in nest structure among individual builders. It seems plausible that textural patterns in other animal constructions may also be more useful for identifying individuality in those structures than the more commonly used measures such as gross morphology. Texture analysis may also allow investigation of variation in structures built by animals from different regions and populations.

Adjusting nest morphology to, for example, differences in climate or position could allow weaverbirds to weave nests that are more suitable to prevailing conditions than a ‘standard’ nest would be. Gross morphology may be determined, therefore, more by a response of a builder to environmental conditions than by individual technique or building style. Indeed, Hawaiian honeycreepers *Hemignathus virens virens*, nesting at higher altitudes do build nests with thicker, better insulated walls than do those honeycreepers nesting at lower altitudes [46]. It is not clear, however, whether adding nest material has much impact on any individual structural features, or the way in which male weavers weave materials.

It is also possible that an individual's weave pattern becomes inflexible after an initial learning period, similar to song learning in some species [47], or that individuals are inflexible in their weave pattern as it is genetically predetermined, restricting within-male variability in weave pattern. This second possibility seems unlikely as nest weaving is partly a learnt behaviour [17,48].

Correct classification success using our textural analysis approach declined as the number of nests per male included in the analysis increased. This was particularly evident for the southern masked weaver data: the 2009 dataset included on average 3.29 nests per male, while the 2008 dataset included on average 5.14 nests per male (table 2). The classification results were 81.82% and 33.33%, respectively. Although based on just four datasets, a lower classification accuracy in datasets containing more nests per male was not unexpected as including more nests and more males into an analysis increases the opportunity for errors (e.g. there is greater opportunity for inclusion of nests built under unusual environmental conditions and therefore atypical of the building style of that male). It is also possible that as more males were included in a dataset, the greater the chance that two or more of them had a very similar nest-weaving pattern.

It is not possible to discern from this analysis what it is about texture of the nest surface that differs among males as texture analysis methods do not provide relevant information. Texture analysis just allows confirmation or not of individuality and signatures. That we have detected consistent individual differences among males in the texture of the nests they build tell us that there were either physical or behavioural difference among males that influenced the nests they built. This variation among males may affect their survival and reproduction, particularly when nest texture correlates with features of a nest's structural properties. Detailed behavioural analyses and biomechanical testing of the integrity of nests might be a useful next step to investigate this possibility.

Among the classification methods we explored, wavelet transform appeared to be the most useful, as descriptors computed using it were selected as part of the best classification approach in all datasets. The frequent selection of wavelet transform in the best classification approaches was expected as this method presents some advantages in signal/image analysis in comparison to other methods [28,39,41].

In summary, we have found that computer-aided textural analysis methods, in particular the wavelet transform approach, will correctly attribute weaverbird nests to the individual that built them. Importantly, this approach appears to be able to identify a signature to individual building styles not seen using the traditional gross-morphology measures of animal structures. Texture analysis approaches might, then, be applied usefully to structural analysis of other animal constructions.

## Data accessibility

All images used in this study are available from the Dryad Digital Repository: http://dx.doi.org/10.5061/dryad.0t10j.
